# A qualitative synthesis of factors influencing maintenance of lifestyle behaviour change in individuals with high cardiovascular risk

**DOI:** 10.1186/1471-2261-13-48

**Published:** 2013-07-06

**Authors:** Jenni Murray, Grania Fenton, Stephanie Honey, Ana Claudia Bara, Kate Mary Hill, Allan House

**Affiliations:** 1 Academic Unit of Psychiatry and Behavioural Sciences, Leeds Institute of Health Sciences, The University of Leeds, Charles Thackrah Building, 101 Clarendon Road, Leeds LS2 9LJ, UK

**Keywords:** Cardiovascular diseases, Lifestyle, Health behaviours, Primary prevention, Secondary prevention, Primary health care, Maintenance

## Abstract

**Background:**

Management of cardiovascular risk factors includes commitment from patients to adhere to prescribed medications and adopt healthy lifestyles. Unfortunately many fail to take up and maintain the four key healthy behaviours (not smoking, having a balanced diet, limiting alcohol consumption and being more active). Five factors (beliefs, knowledge, transport and other costs, emotions, and friends and family support) are known to predict uptake of lifestyle behaviour change. The key factors influencing maintenance of healthy lifestyles are not known but would be helpful to support the development of relapse prevention programmes for this population. Our review aimed to clarify the main patient perceived factors thought to influence maintenance of changed healthy lifestyles.

**Methods:**

We performed a systematic review of qualitative observational studies and applied the principles of content synthesis and thematic analysis to extract reported factors (barriers and facilitators) considered by individuals to be influential in maintaining changed healthy lifestyle behaviours. Factors were then organised into an existing framework of higher order categories which was followed by an analysis of the interrelationships between factors to identify key themes.

**Results:**

Twenty two studies met our inclusion criteria. Participants reported barriers and facilitators within 13 categories, the majority of which were facilitators. The most commonly reported influences were those relating to social support (whether provided formally or informally), beliefs (about the self or the causes and management of poor health, and the value of maintaining lifestyle behaviours), and other psychological factors (including attitude, thinking and coping styles, and problem solving skills). Physical activity was the most commonly investigated behaviour in four categories, but overall, the main barriers and facilitators were related to a range of behaviours. Through analysis of the interrelationships between factors within categories, ‘social support’, ‘education and knowledge’, and ‘beliefs and emotions’ were all considered key themes.

**Conclusions:**

Our review suggests that for the most part, factors that influence lifestyle change are also important for maintaining healthy behaviours. This indicates that addressing these barriers and facilitators within lifestyle support programmes would also be of value in the longer-term.

## Background

Evidence supporting the role of healthy lifestyles in the prevention and management of a range of long-term conditions including cardiovascular disease is compelling [1,2]. Despite this, many individuals struggle to take up and maintain healthy lifestyle behaviours. Uptake for cardiac rehabilitation, for example, is poor (44%) [3] and there are high attrition rates for commercial weight management programmes [4]. For those who are successful in making changes, relapse rates are high. For example, over 75% of quitters return to smoking within one year [5,6], and 50% of dieters regain lost weight after one year [7]. Poor uptake rates can be attributed to a myriad of social, psychological and practical barriers that are challenging to address within healthcare consultations. However, by focusing on a few factors that are known to predict uptake (beliefs, knowledge, transport and other costs, emotions, and support from family and friends) [8,9], practitioners can start to consider the most appropriate type and level of support for each individual. As with uptake, there are likely to be a range of factors that influence an individual’s ability to maintain their healthy behaviours. Knowledge of the main barriers and facilitators that influence maintenance of healthy lifestyles could be used by formal programmes to develop more effective behavioural relapse prevention interventions.

Much of the theory and evidence informing relapse prevention strategies comes from the addictions literature in relation to alcoholism, smoking and obesity [10-12], with comparatively little relating to physical activity and poor diet. Whilst all these behaviours are important in the context of reducing cardiovascular risk, we hypothesise that the types of barriers and facilitators and thereafter the relapse prevention strategies observed in the addictions literature may not be fully appropriate or comprehensive for individuals at high risk of cardiovascular events (including diabetes, hypertension, and hypercholesterolemia). Many patients in this category will need to change and maintain multiple lifestyle behaviours that involve diet modification, alcohol reduction and increased physical activity levels, with clinical aims and benefits that may be less tangible to the lay person. This is particularly the case for those who have been given a prospective cardiovascular risk score through screening, a common practice in primary care in many developed countries [13-16]. Further, this particular population may perceive more severe consequences and greater levels of fear associated with failure of lifestyle change compared to individuals with, for example, obesity in the absence of other cardiovascular risk factors. Finally, lifestyle management of individuals at high risk of cardiovascular events may not necessarily involve provision of statutory services such as smoking cessation or obesity weight management, and rates of relapse might be different than in those who change their behaviours without formal support [12]. Given these uncertainties, we considered it important to conduct a thorough review of the literature to elicit the main factors that influence maintenance of lifestyle behaviour change in individuals at high risk of cardiovascular events. Our ultimate aim is to inform the development of relapse prevention interventions that are relevant to these particular populations.

## Methods

### Selection criteria

We included empirical qualitative observational studies reporting factors related to the maintenance of specified lifestyle behaviours (diet for weight loss or healthy eating purposes, alcohol consumption, smoking and physical activity). Participants were adults (≥18 years) who: were previously or currently obese (BMI ≥30); experienced angina, myocardial infarction or transient ischemic attack or; were living with coronary artery disease, chronic obstructive pulmonary disease(COPD), hypertension, hyperlipidemia, metabolic syndrome or type II diabetes. Studies were excluded if they focused on a selected population such as tribal groups or people with mental health difficulties.

Given the range of definitions of maintenance for the numerous healthy lifestyle behaviours [17-22], and the lack of clear consensus on which is most appropriate, we adopted a flexible approach to guide study selection and data extraction. Studies were required to demonstrate that at least some of the participants had, at some point in the past, successfully made changes to their lifestyle and that any current attempts to maintain the changed behaviours were without any on-going formal support programme, such as, for example, weight management or smoking relapse prevention support. However, studies involving those attending a local leisure centre by their own volition and those receiving ongoing support as part of their condition (i.e. attending a diabetes clinic) would be included. Studies including data on both changing and maintaining lifestyles needed to report separate findings for each.

### Searching

An electronic search strategy was developed (available on request from authors) and run (November 2011) in Embase, Medline, PsycInfo, Applied Social Sciences Index and Abstracts (ASSIA), Allied and Complimentary Medicine (AMED) databases. Key search terms were also applied in Cumulative Index to Nursing and Allied Health (CINAHL), and all databases were searched from 1970 onwards or from database inception if more recent. Search results (managed in Endnote, Version X5) underwent title, abstract and full paper screening by at least two independent reviewers (JM, GF, SH, ACB) against pre-defined selection criteria. Disagreements about inclusion were referred to a third reviewer, and all were resolved. Personal contact was made with authors of several papers to clarify details necessary for determining inclusion. Non-response led to exclusion.

### Data extraction: identification of factors

Data extraction of factors reported in included studies was performed independently by at least two reviewers (SH, GF, JM, ACB) followed by consensus checking. We extracted those factors demonstrating consensus between participants and deemed them relevant if they were reported as helping (facilitators) or hindering (barriers) maintenance of healthy lifestyle behaviours. Each factor was entered as an individual record into a Microsoft Excel 2007 spreadsheet along with contextual data relating to the health condition and behaviour(s) under investigation, and facilitator or barrier status.

### Data analysis: aggregation of factors into categories

Through an iterative process involving interpretation and consensus (GF & JM), we then systematically applied principles of content synthesis and thematic analysis [23,24]. This involved a detailed analysis of extracted factors and their interrelationships, and, wherever possible, organising extracted factors to an existing framework of higher order categories [25]. As the previous framework was derived from literature that primarily focused on changing behaviours, extracted factors that did not fit were grouped and assigned to new categories.

### Identification of key themes

**Figure 1:**
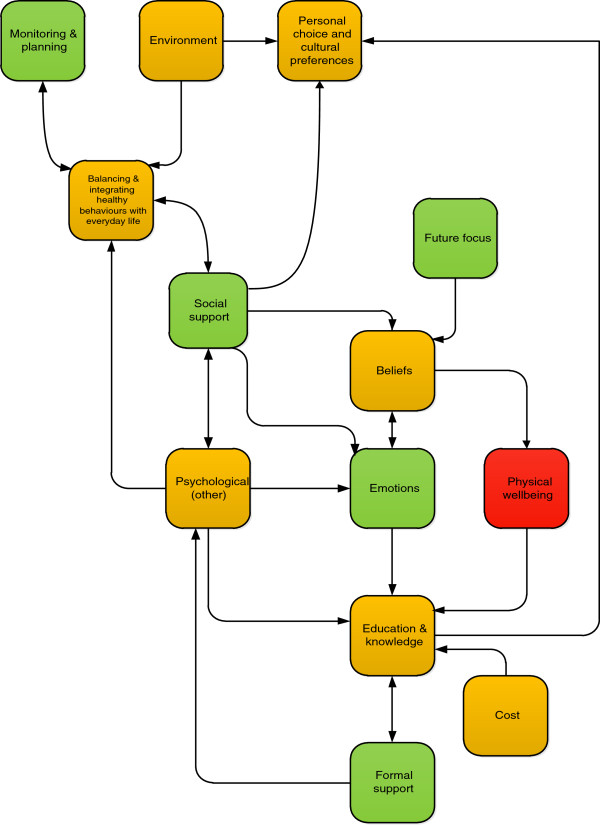
**Relationships between categories of identified facilitators and barriers to the maintenance of changed lifestyle behaviours.** Green boxes represent categories in which 60% or more factors were facilitative. Red represents categories which 60% or more factors were barriers. Orange represents categories with a relatively balanced mix of barriers and facilitators. Unidirectional arrows indicate that factors in one category related to another (e.g. thinking about the future was related to beliefs about the benefits of healthy lifestyles). Bidirectional arrows indicate that factors in both categories made links with each other.

Two reviewers (GF & JM) simultaneously reviewed individual factors within each category to identify links with other categories. Links were then used to create a relationship map (Figure 1). JM and GF then examined the map to identify categories that might represent key themes. This involved identification of the categories that appeared to occupy a central role in the map, having multiple links to and from other categories. Thereafter, judgements were made about which of these categories might usefully inform the core components of a relapse prevention intervention.

### Quality assessment

The quality of the studies was assessed using a 36-item tool [26,27]. The tool primarily comprised items from the widely used COREQ [26] with additional questions that were considered important from Long & Godfrey [27]. Each item scored 2, 1, or 0 representing the extent to which each criterion was met, with a maximum possible score of 72. We applied arbitrary cut-offs to categorize studies as good (>65%), fair (35%-65%) and poor (<35%), and used this as a descriptive tool rather than exclusion criterion.

## Results

The electronic search strategy identified 15,731 publications and following title and abstract screening, 332 full papers were obtained. Of these, 309 were rejected because they: reported quantitative data only (n=248); were not empirical (n=19) or a peer reviewed paper (n=1), or did not meet other inclusion criteria, including a lack of focus on the target population or not reporting maintenance related themes (n=41). Included in the review were 22 studies, reported in 23 papers [28-50].

### Summary of the studies

The studies recruited 723 participants (Table 1). The majority of studies were conducted in the UK (n=10; 45%), with 41% (n=9) in the USA, two in Taiwan and one in Australia. Most participants belonged to the majority ethnic group of their country, and most studies collected data through individual interviews (n=15). The conditions under investigation were Type 2 diabetes (n=7), heart disease (n=6), obesity or COPD (n=3 each) hyperlipidemia (n=2) and hypertension (n=1). More than half of the studies (n=15) focussed on two or more lifestyle behaviours, with physical activity being the most often investigated behaviour (20 studies) and alcohol intake the least (one study) [34]. The shortest period of maintenance was within one month following completion of an intervention [40] with 46 years’ post-diagnosis being the longest [28]. The majority of studies focused on a possible maintenance period of up to two years following a particular event (either diagnosis or onset, treatment or programme participation).

**Table 1:** Characteristics of included studies (n=22)

**Study (in publication date order)**	**N**^**a**^	**Age range**^**b**^	**Risk factor**^**c**^	**Lifestyle behaviours**^**d**^	**Time**^**e**^
Sullivan et al., USA [48]	10	47-77	Type 2 diabetes	Diet, physical activity	at least one year^1^
Gullanick et al., USA [38]	45	34-74	Heart disease	Diet, weight loss, physical activity and smoking	3-18 months^2^
Parry et al., Scotland [44]	48	65-84	Heart disease	Smoking	NR^3^
Byrne et al., England [30]	76	20-60	Obesity	Diet, physical activity	at least one year^4^
Bidgood et al., England [29]	18	NR^f^	Obesity	Diet, weight loss, physical activity)	NR^f^ (all currently obese)^3^
Davis et al., USA [35]	27	30-55	Obesity	Diet, weight loss, physical activity	NR^f^ (all currently obese)
Gregory et al., Scotland [37]	45	Under 65 years (NOS)^g^	Heart disease	Diet, physical activity and smoking	2-3 years^5^
Nagelkerk et al., USA [42]	24	26-78	Type 2 diabetes	General including diet	Range 1–26 years (mean 9.93)^1^
Dailey et al., USA [33]	23	40-77	hyperlipidemia	Diet, physical activity	within one year^1^
O’Shea et al., Australia [43]	22	51-79	COPD	Physical activity	12-24 weeks^6^
Lee et al., Taiwan [39]	22	All aged > 60 years (NOS^g^)	hypertension	Physical activity	within one month^6^
Chen et al., Taiwan [31]	18	55-81	COPD	Diet, physical activity, smoking	at least one year^3^
Darr et al., England [34]	65	40-83	General	Alcohol, diet, physical activity, smoking	within one year^7^
Coghill et al., England [32]	38	54.8 mean (SD 5.0)	hyperlipidemia	Physical activity	12 weeks^6^
Gazmararian et al., USA [36]	24	56 (mean:no SD)	Type 2 diabetes	Diet, weight loss, physical activity	at least 6 months^1^
Malpass et al., England [41]	30	30-80	Type 2 diabetes	Diet, physical activity	12-18 months^1^
Peel et al., Scotland [45]	21	NR	Type 2 diabetes	Physical activity	6-12 months^1^
White et al., England [49,50]	15	42-72	Heart disease	Diet, smoking, physical activity	9 months^6^
Lewis et al., England [40]	6	61-83	COPD	Physical activity	at least one month^6^
Peterson et al., USA [46]	61	46-86	Heart disease	Diet, weight loss, smoking, physical activity	at least one year^4^
Beverly et al., USA [28]	60	51-81	Type 2 diabetes	Physical activity	1-46 years^1^
Rahim-Williams et al., USA [47]	25	46-87	Type 2 diabetes	Diet, weight loss, physical activity	3-41 months^1^

^a^ this is the number of participants recruited to the study, not the number from whom data were extracted.

^b^the age ranges of participants entering the studies was extracted where possible. For those studies where this was not reported, the mean, and if reported, standard deviation, were also extracted.

^c^this is the primary risk factor reported within the study, though participants in many had more than one.

^d^ we did not necessarily extract data relating to all lifestyle behaviours: maintenance related themes were our primary focus.

^e^time since: 1 = diagnosis; 2 = surgery alone; 3 = onset (where time since formal diagnosis not reported); 4 = maintenance of lifestyle behaviours; 5 = discharge from hospital; 6 = post-intervention, and; 7 = diagnosis or surgery.

^f^NR = not reported.

^g^ NOS = not otherwise specified.

### Factors, categories and key themes

A total of 97 factors (1–11 per study) were extracted and organised into 13 categories (Table 2). Six of the categories were those from our previous framework [25], five were modified (to better reflect the range of factors in the current review), and the remaining two emerged from the extracted data (‘future focus’ and ‘monitoring and planning’). Unlike the previous review that reported the majority of factors as barriers to lifestyle behaviour change [8], most of the factors in the current review were deemed to be facilitators (n=64; 66%). The category containing the greatest proportion of barriers was ‘physical wellbeing’. The largest category (containing the most factors) was ‘social support’ followed by ‘psychological (other)’ and ‘beliefs’ (Table 3). Two studies with a particular focus in these areas contributed substantially to these categories [28,30]. However even without their contribution, these types of factors would remain the most common. No other categories were excessively influenced by any one study.

**Table 2:** Summary description of factors within each category

**Category**^**a**^	**Description of factors within each category**
Social support‡	Support provided informally by friends and family, or peers within a group, whether that group was selected by the individual, or one to which they were referred. Primarily facilitative and relating to physical activity. The presence of another with whom participants could be active, or who could adapt alongside them or encourage them, was reported as particularly beneficial.
Psychological (other)	Primarily facilitative, encompassed psychological factors such as attitude, motivation, confidence, determination, persistence, thinking and coping styles and problem solving skills, as well as self- identity.
Beliefs*	Beliefs about self, the causes and management of poor health, and the value of maintaining lifestyle changes, in addition to spiritual beliefs. Largely facilitative.
Formal support^‡^	Two types of support: formal support in general, or specific to the types of support individuals would like from a healthcare professional. Support from a healthcare professional included supervision and monitoring and advice for individuals or family members. Barriers included a perceived lack of co-ordinated care, whereas facilitators include a relationship that provided education as well as positive feedback.
Balancing and integrating healthy behaviours with everyday life	Connected to participants’ other commitments, routines and time.
Emotions	Positive facilitative emotions such as a sense of pleasure, achievement or satisfaction, those reported as a barrier or facilitator (e.g. fear), and those reported as barriers alone, including stress or a sense of frustration.
Physical wellbeing	Primarily barriers (all to physical activity) including co-morbidities and injuries.
Education and knowledge	Education typically related to formal support, or to knowledge gained less formally. Facilitators in relation to dietary knowledge, and barriers relating to up to date knowledge in preparation for, and during, maintenance.
Environment	Mainly about the weather, but also incorporated exercise venues as a barrier, an enjoyment of nature and using music as a distraction. All relating to physical activity.
Monitoring and planning†	Participants’ specifications that the monitoring (e.g. of weight) and planning (e.g. of meals and goals) were facilitative to the maintenance of lifestyle behaviour changes.
Personal choice and cultural preferences	Facilitators related to the variety of exercise options and resources available to participants, barriers to managing a healthy diet and weight related to cultural gatherings, related foods and expectations.
Cost	Costs associated with leisure facilities and healthier foods.
Future focus†	Only recorded as a facilitator, this referred to motivation driven by future goals, including spending time with family members.

a-Categories are either those that: were reported and have remained essentially unchanged (unmarked) from previous framework [25]; have been created by amalgamating categories (e.g. friends and family support combined with social support into social support) from previous framework ‡; were the result of splitting previous categories; or new categories †.

**Table 3:** Categories, key themes (bold and italicised) and factors

**Categories**	**Number of studies**	**Factors**					
		** All**		** Barriers**		** Facilitators**	
		***n***	***%***	***n***	***%***	***n***	***%***
***Social support***	12	19	19.4	4	21	15	79
Psychological (other)	10	16	16.3	4	25	12	75
Beliefs*	10	11	11.2	4	36	7	64
Formal support	7	9	9.3	4	44	5	56
Balancing and integrating healthy behaviours with everyday life	6	8	8.2	4	50	4	50
***Emotions***	7	7	7.1	2	29	5	71
Physical Wellbeing	6	6	6.1	5	83	1	17
***Education and knowledge***	5	5	5.1	2	40	3	60
Environment	4	5	5.1	2	40	3	60
Monitoring and planning	4	4	4.1	0	0	4	100
Personal choice and cultural preferences	3	3	3.1	1	33	2	67
Cost	2	2	2.0	1	50	1	50
Future focus	2	2	2.0	0	0	2	100
All	*Range 2-12*	97		33		64	

*The beliefs category incorporated 11 papers reporting 10 separate studies.

In relation to the distribution of behaviours across the categories, physical activity dominated ‘social support’ and ‘balancing and integrating healthy behaviours with everyday life’, and was the exclusive behaviour in ’physical wellbeing’ and ‘environment’. All other categories covered a broader range of behaviours.

We identified four potential key themes (Figure 1). Factors in ‘social support’ related to five other categories. In turn two other categories also made links back to ‘social support’. Its position within the relationship map demonstrates that social support may be the main gatekeeper to ‘balancing and integrating healthy behaviours with everyday life’ which in turn links to ‘monitoring and planning’ and ‘environment’. This category was therefore considered a key theme. Examination of the ‘education and knowledge’ category revealed that the five factors within this made reference to two other categories (‘formal support’ and ‘personal choice and cultural preferences’). In turn, factors within ‘formal support’ and four other categories (‘psychological (other)’, ‘emotions’, ‘physical wellbeing’, and ‘cost’ made reference back to ‘education and knowledge’. Thus ‘education and knowledge’ seems to be at the core of many factors and this category was considered a key theme. Finally, ‘beliefs’ also represented a large category and although it might be influenced by good social support, its influence on a number of other categories and the specific nature of some of the factors (relating to poor self-belief) suggest that it may be a key theme. Closely linked with this category is ‘emotions’, which if presented as depression or anxiety may be a significant barrier to maintenance and hence require specific attention. Emotions could therefore be combined with beliefs to act as a key theme.

### Quality assessment

Two papers were ‘poor’ in quality [39,44], 20 were ‘fair’ and one was ‘good’ [38]. The median score was 44 (51%, range 26–60). Studies were most likely to score well on reporting the background to the study and aspects of the study design, including their use of an analytic framework, methods of data collection and description of theme generation (Table 4). They scored least well in providing details about characteristics of the research team, relationships between the researchers and participants, information about the presence of others during data collection and whether themes were validated with participants.

**Table 4:** Summary of quality assessment of 23 included papers (reporting 22 included studies)

**Section and sub-section of tool**	**No. items**	Item most often addressed (number of studies*)	Item least often addressed (number of studies*)
Background	1	Is it clear what is being studied? (23)	N/A - only one item in this sub-section
Research team and reflexivity			
*Personal characteristics*	5	Is the gender of the researcher clear? (15^†^)	Were the characteristics of the interviewer reported (bias, assumptions, reasons and interests in the topic? (1^†^)
*Relationships established*	2	Is there evidence that the researcher/interviewer had any informal contact with the participant before the study commenced (i.e. ‘chats’)? (4^†^)	Did the researcher/interviewer indicate if there was a previous therapeutic or personal relationship with the participant and if so, was this described? (1^†^)
Study design			
*Analytic framework*	1	Was use of an analytic framework mentioned (e.g. grounded theory, discourse analysis, ethnography phenomenology, content or thematic analysis? (19^†^)	N/A - only one item in this sub-section
*Participant selection*	4	Does the study state how many took part in the interviews? (23)	Does the study state how many refused or dropped out and does it provide reasons? (10^†^)
*Setting*	3	Are the relevant characteristics of the sample reported (demographics)? (22^†^)	Does the researcher state if anyone else was present during the interviews? (8)
*Data collection*	7	Does the author say how many interviews were carried out? (23)	Does the study state if supplementary field notes were made during/after the interview or focus groups (9) and was data saturation discussed? (9^†^)
Data analysis and findings			
*Data analysis*	6	Does the author state if themes were identified in advance or from the data? (23)	Did the authors report checking back with informants over interpretation? (6)
*Reporting*	5	Were major themes clearly presented in the findings? (23^†^)	Are all participant quotations labelled according to participant? (12^†^)
Ethics	2	Was informed consent obtained from all study participants? (17^†^)	Does the study report if ethical approval was obtained? (14^†^)

*The number of studies completely or partially addressing each item.

^†^ (Some studies partially addressed this item).

## Discussion

To our knowledge, this is the first systematic review of the qualitative literature reporting the main patient perceived factors that influence maintenance of changed healthy behaviours in individuals at high risk of cardiovascular events. Information such as this is important because maintenance rates of changed healthy behaviours in this particular population diminish with time [30,50] and lifestyle support programmes that do not deal with addictions are unlikely to offer evidence-based relapse prevention interventions [51]. Highlighting the key factors that patients consider important in maintaining their healthy behaviours will be needed to support the development of such interventions.

### Comparison with existing literature

Although some individuals change and maintain their healthy behaviours with relative ease and little support, many undergo a series of steps (often termed stages of change [17]), that can be non-linear, involving contemplation, action and maintenance. Maintenance therefore represents the tail end of a continuum of change and so should not be viewed in isolation. Previous reviews examining factors associated with changing lifestyles in this population found that low mood, misplaced beliefs about the causes and value of healthy lifestyles, poor knowledge, limited social support (from friends and family) and difficulties with transport and related costs predicted non-uptake of lifestyle behaviour change and non-completion of related programmes [9]. The current review seems to suggest that for the most part, the areas that influence change of lifestyle behaviours also influence maintenance. Furthermore, as with change, the influencing factors are interlinked. The literature, however, only offers us the factors and as such we cannot explain the nature of the links or how they may differ between individuals or perhaps types of individuals. Insight into this might offer a clearer explanation as to why some maintain and others do not. Based on our current knowledge, we at least have some guidance as to a core framework that would inform the development of approaches for improving uptake and participation of lifestyle behaviour change, as well as maintenance. This would be aided by a more detailed comparison of the factors relating to change and maintenance to ensure that programmes provided relevant support for each stage.

### Summary of main findings

We considered at the outset that maintenance of changed lifestyle behaviours, as reported in the cardiovascular literature, would potentially highlight some differences from the addictions literature. As predicted, we have observed that individuals are attempting to maintain multiple changed lifestyles, though the literature failed to describe particular challenges associated with their simultaneousness. Further, physical activity was the most commonly investigated behaviour in this review, while sedentary lifestyles are not addressed in the addictions literature.

Social support was identified as a key theme in the current review. Also a key theme in the active stage of changing lifestyle behaviours [8], its continued importance in the longer-term is unsurprising. This is a challenging area to address where social support is lacking. In order to facilitate maintenance of changed behaviours, good social support will be needed much earlier on to ensure that beliefs about the benefits of healthy lifestyles are more stable and effective planning and problem solving are in place to support continued integration with everyday life. Evidence suggests that whilst changing behaviours with a friend is more likely to be successful [52-54], this is not sustained where obesity and overweight are the norm in the broader social network [55,56]. A more creative approach to tackling obesity through a predominantly social model may therefore be required.

The ‘beliefs’ core theme comprised mostly barriers and to some extent this was unexpected. Given that the included studies comprised individuals who had attempted to make changes to their lifestyle (presumably because of their conditions), it was assumed that beliefs about the value of a healthy approach to life would be relatively stable. However there was evidence that individuals were still questioning the benefits of healthy lifestyles and we can only speculate about the reasons why. Changes to lifestyle may have been triggered by an event or diagnosis. However, if clinical outcomes (e.g. blood pressure, cholesterol, HbAC1 levels) do not respond as anticipated, or perceptions of risk alter through time (due to, for example, absence of subsequent events), beliefs may be challenged. Clearer information about the impact of changing behaviours on cardiovascular risk would be beneficial, particularly given that patients tend to underestimate their personal risk [57]. Currently Framingham and QRISK, algorithms for calculating cardiovascular risk [58,59] are used routinely in primary care, and their associated software enables visual adjustment of risk scores according to behaviour for one variable, smoking. This is not currently available for diet, physical activity and alcohol, although should be possible with good epidemiological data such as that reported for multiple lifestyle factors in the prevention of cerebrovascular events [60]. Development of a decision aid for lifestyle behaviours, such as that used for statin prescribing in the UK [61] would be of value. However, training in their use would be required, as health care professionals tend to avoid discussions of risk that incorporate visual and numerical framing [57,62].

Some of the factors were reported as strategies (facilitative thoughts and actions) that individuals applied whilst trying to maintain healthy diets and physical activity. These included planning meals, getting into a routine and self-monitoring, all of which are typical of the cognitive behavioural and problem solving approaches that form the basis for relapse prevention programmes [10,11]. Evidence suggests that use of such strategies are common amongst successful abstainers of weight loss and smoking [17,63]. In our review, only one facilitative factor (formation of a new identity) [44] was specifically related to maintenance of smoking cessation, but in the absence of further detail it cannot be considered a strategy. Five studies [32,40,43,49,50,64] explicitly stated that participants had previously been in a formal lifestyle programme. There is no suggestion from these that individuals were given any relapse prevention training and indeed from these studies there were numerous examples of barriers. The facilitative strategies observed across the studies may therefore have been acquired by chance rather than through any formal support mechanism.

### Strengths and limitations

The studies included in the current review were individually small, as is typical for qualitative research however collectively they form a substantial evidence base.

None of the included studies defined what they meant by maintenance and therefore we had to make subjective judgements about factors that appeared to be relevant. To some extent we reduced the risk of reporting factors about ‘changing’ behaviours by excluding studies in which the participants were actively involved in or had just recently completed a formal lifestyle programme. Nonetheless, extra diligence was required during data extraction to ensure that inadequately described retrospective data on the change process in included studies was omitted. Factors that were particularly challenging included those that appeared to be about preparation for maintenance (having the right knowledge to continue by oneself with the healthy lifestyle behaviour, and making the ‘right personal choice’ of venue for exercising). These have been reported in this review.

As with factors, decisions on key themes were somewhat subjective. The process did however involve two reviewers and take into account the number of links to and from categories. The only category that lacked clarity as to whether or not it represented a key theme was ‘psychological (other)’. Our interpretation is only a guide and we suggest that the development of any associated intervention for relapse prevention also considers individual attitudes, motivations and confidence.

All but two studies appeared to report inductively generated results that were independent of their stated aims [28,30]. These studies contributed substantially to three categories: ‘social support’, ‘psychological (other)’ and ‘beliefs’, although in their absence these categories would have remained large. From this perspective any bias appears to have been limited. We cannot however, rule out the possibility that the authors themselves may have held particular philosophical viewpoints that may have biased the collection or analysis of the original data.

Apart from maintained weight loss, the studies did not indicate that any objective measures had been used to demonstrate continued abstinence of unhealthy behaviours. We were therefore reliant on the truthfulness of the participants within the studies. Many of the studies examined factors relating to maintenance of a healthy diet (for their condition rather than for weight loss) and physical activity. As none of the studies indicated whether these activities met with existing recommendations, we might assume that some individuals, although possibly unaware, were not fully compliant. This being the case, perceptions about the ease with which healthy behaviours are maintained may bias the findings towards facilitators. This might explain why we observed more facilitators than barriers in the studies.

## Conclusions

Social support, education and knowledge, and beliefs and emotions are key areas that lifestyle support services need to focus on within the context of facilitating lifestyle behaviour change and maintenance. While further confirmation about their role in predicting maintenance of changed behaviours is required, it remains likely that these key areas are those that collectively appear to provide the framework that binds changed behaviours to everyday living. In delivering this type of support there needs to be better integration between health and social care. In the UK there is a large strategic shift towards better preventive services through the creation of Public Health England, the core of which comprises health and social care professionals working within Integrated Health and Wellbeing Boards [65] to commission local services according to needs. The bringing together of these very different philosophical stances will bring challenges but also opportunities to more effectively tackle the unhealthy consequences of societal problems.

## Competing interests

The authors declare that they have no competing interests.

## Authors’ contributions

JM wrote the study protocol and led the review process and writing of the manuscript. GF was involved in all stages of conducting the review, including screening, data extraction and the writing of the manuscript. SH was involved in screening and data extraction and reviewed the manuscript. ACB was involved in screening and reviewed the manuscript. KH reviewed the study protocol and the manuscript. AH conceived the idea for the review and reviewed the study protocol and the manuscript. All authors have read and approved the final manuscript.

## Authors’ information

Jenni Murray, BSc, MSc, Phd, Senior Research Fellow

Grania Fenton, BA, PGCert, DClinPsychol, Research Fellow

Stephanie Honey, BSc, MSc, Phd, Research Fellow

Ana Claudia Bara, BSc, BA, PhD, Research Fellow

Kate Hill, BSc, MSc, Phd, Senior Research Fellow

Allan House BSc. MBBS, MRCP, MRCPsych DM, Professor of Liaison Psychiatry.

## Pre-publication history

The pre-publication history for this paper can be accessed here:

http://www.biomedcentral.com/1471-2261/13/48/prepub
